# Bacteria Associated with *Copestylum* (Diptera, Syrphidae) Larvae and Their Cactus Host *Isolatocereus dumortieri*

**DOI:** 10.1371/journal.pone.0027443

**Published:** 2011-11-23

**Authors:** Ana Paola Martínez-Falcón, Ana Durbán, Amparo Latorre, Josefa Antón, María de los Ángeles Marcos-García

**Affiliations:** 1 Instituto de Biodiversidad CIBIO, Universidad de Alicante, Campus Universitario San Vicente del Raspeig, Alicante, España; 2 Unidad mixta Instituto Cavanilles de Biodiversidad y Biología Evolutiva (Universidad de Valencia) y Centro Superior de Investigación en Salud Pública (Generalitat Valenciana), València, España; 3 Departamento de Fisiología, Genética y Microbiología, Universidad de Alicante, Campus Universitario San Vicente del Raspeig, Alicante, España; Argonne National Laboratory, United States of America

## Abstract

We describe the gut bacterial diversity inhabiting two saprophagous syrphids and their breeding substrate (decayed tissues of the columnar cactus *Isolatocereus dumortieri*). We analyzed the gut microbiota of *Copestylum latum* (scooping larvae that feed on decayed cactus tissues) and *Copestylum limbipenne* (whose larvae can also feed on semiliquid tissues) using molecular techniques. DNA was extracted from larval guts and cactus tissues. The V1-V3 region of the 16S rRNA genes was amplified and sequenced. A total of 31079 sequences were obtained. The main findings are: *C. limbipenne* is dominated by several Enterobacteriaceae, including putative nitrogen-fixing genera and pectinolitic species and some denitrifying species, whereas in *C. latum* unclassified Gammaproteobacteria predominate. Decayed tissues have a dominant lactic acid bacterial community. The bacterial communities were more similar between larval species than between each larva and its breeding substrate. The results suggest that the gut bacterial community in these insects is not strongly affected by diet and must be dependent on other factors, such as vertical transmission, evolutionary history and host innate immunity.

## Introduction

**Figure 1:**
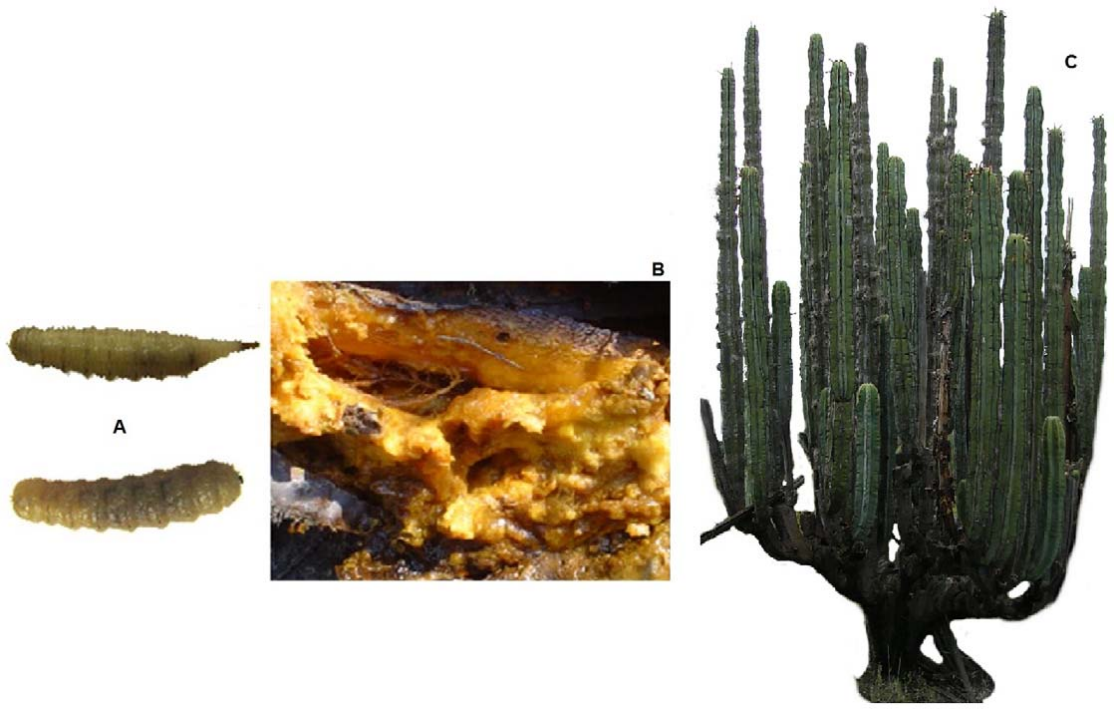
Larval species (a), decayed cactus tissues (b) and columnar cactus *Isolatocereus dumortieri* (c).

*Copestylum* is a neotropical endemic syrphid lineage that harbours one of the highest species richness, with over 400 species [1], [2], [3]. Larvae of *Copestylum* are saprophagous (Figure 1) and live in a large variety of microhabitats, with decaying Cactaceae and Agavaceae tissues as one of their most frequently reported breeding media [4], [5]. Saprophagous syrphids are ecologically important because of the potential role of their larvae in nutrient recycling processes [3], [6], [7]. For instance, larvae of *Copestylum* Macquart 1986 (Diptera: Syrphidae) are commonly bred in decayed cactus species and assist in the degradation of cactus necroses contributing to recycling processes in xeric environments [6], [7], [8].

Besides their ecological importance, this group presents interesting feeding strategies: Rotheray *et al.* found morphological differences among *Copestylum* larvae reared from Cactaceae [3]. They found two functional morphological trends: one trend is towards feeding on watery decay and the other towards feeding in firmer decay. The species that can feed on solid material have specialized grinding mills in their head skeletons to break up the tissues and scoop food, specialized armoured thoraces for gripping and protection during tunnelling, and a short posterior breathing tube. The species that can feed on watery material (straining) have reduced armature and have an elongate posterior breathing tube. The elongate breathing tube in *Copestylum* species enables them to obtain atmospheric oxygen from these decomposed substrates. Finally, some species are intermediate between these feeding strategies. Examples of scooping species are *C. latum* and *C. posticum*; some straining species are *C. mila* and *C. hidalgense*, and the intermediate species are *C. limbipenne* and *C. marginatum*

There are no studies about the microbial community found in the intestinal tract of *Copestylum* larvae. Otherwise, the roles of microorganisms are well-studied in the cactus-microorganism-*Drosophila* model [9], [10]. Bacteria are the first microorganisms to grow in newly injured tissue, cactophilic yeast are secondary invaders and the medium created by bacteria serve to host selection for *Drosophila* and stimulate oviposition. Bacteria are also important sources of nutrition for larvae.

The ecology of cactus degradation (Figure 1) is a complex process, involving many different interacting microorganisms, including both yeast and bacteria [10]. Arms and stems of columnar cacti (Cactaceae) occasionally become necrotic and serve as feeding and breeding sites for a variety of arthropods [3]. Several kinds of these rots develop when bacteria and yeast colonize tissue weakened by injury, environmental stress or senescence. The bacterial communities utilizing the necrotic tissues of columnar cacti are important components of the decayed tissues; injured cactus tissue can be infected by bacteria in the environment developing a rot pocket or necrosis [11]. In cactus necrosis, microbes lyse the plant cells, creating a wet, nutrient-rich microenvironment in the midst of the xeric environments. Necrosis provides substrates for feeding and breeding to cactophilic species such as beetles (Coleoptera) [12] and flies (Diptera) [13].

*Isolatocereus dumortieri* (Scheidw) Backeb (Figure 1) is a cactus species endemic of the central Mexican semiarid scrublands [14], [15]. This cactus is a common breeding medium for hoverflies [6], [7].

The characterization of the interactions in this cactus-microorganism-hoverfly system provides valuable information about host selection and feeding behaviour of the hoverflies in xeric environments, and important data about the role of each component (microorganisms and hoverflies) in decomposition processes in Mexican scrublands. Despite their central role in the cactus-microorganism-hoverfly system, the bacterial component has not been characterized. There is a complete lack of information on the microorganisms inhabiting both decaying cacti and larvae breeding on them, which is a key to understand the interactions developing between the cactus and the insect.

This study describes, for the first time, the bacterial diversity inhabiting in necrotic tissue of the columnar cacti *Isolatocereus dumortieri* and in the gut of two species of *Copestylum* by partial sequencing of 16S rRNA genes directly amplified from samples. We have chosen two species of *Copestylum* with two different feeding behaviours: *C. latum,* which can scoop decayed tissues, and *C. limbipenne,* which has an intermediate behaviour between scooping tissues and feeding on liquid decomposed cactus. The goals are to know whether these two different species of *Copestylum* larvae harbour different microbiota, what the differences in the microbial communities inhabiting cactus tissues in different degrees of decomposition are and to what extent the larval microbiota is related to that of their feeding material. The possible role of bacterial communities in the larval biology and the decomposition of the columnar cactus *I. dumortieiri* are discussed.

## Materials and Methods

### Sample collection

Five samples of decayed cactus tissues from different individuals of *I. dumortieri* (Pap of *Copestylum limbipenne* or PLIM in text) *w*ith larvae of *C. limbipenne* (CLIM) and five different samples of stems of *I. dumortieri* (Pap of *Copestylum Latum* or PLAT in text) with larvae of *C. latum* (CLAT) were collected in one survey in March 2009 in “Barranca de Metztitlán” Biosphere Reserve, Hidalgo, México. In these samples neither species was found together (but other research has reported that they may be found in the same stem of decayed cactus tissue) [6]. All larvae in each sample were collected and placed in 90% ethanol. Necrotic tissue in which each species grew was put in sterile containers that were frozen until further manipulation. Six larvae for each species were randomly chosen from the collected cactus samples for dissection and their complete intestinal tract was extracted using a maculating loop. All necessary permits were obtained for the described field studies. The field studies did not involve endangered or protected species.

### DNA extraction

DNA was extracted from larval guts and cactus tissues as described in Latorre *et al.* [16]. Before DNA extraction, cactus tissues were treated as follows: they were homogeneized in PBS (containing, per litre, 8 g of NaCl, 0.2 g of KCl, 1.44 g of Na_2_HPO_4_, and 0.24 g of KH_2_PO_4_ [pH 7.2]) and centrifuged at 1,800 g for 8 min to remove plant material as far as possible; 1–4 mL of supernatants were centrifuged at 22,000 g for 5 min to pellet bacterial cells.

### PCR amplification of bacterial 16S rRNA gene sequences

DNA samples from each fly species and each cactus tissue were used as templates for PCR amplification of a fragment of the 16S rRNA gene using the composite forward primer 5′-GCCTCCCTCGCGCCATCAGNNNNNNTC*AGAGTTTGATCMTGGCTCAG*-3′ (where the underlined sequence is that of 454 Life Sciences primer A, NNNNNN designates the unique six base barcode used to tag each PCR product, and the broad range bacterial primer B8F is in italics), and the reverse primer 5′-GCCTTGCCAGCCCGCTCAGGC *TGCTGCCTCCCGTAGGAGT*–3′ (where the underlined sequence is that of 454 Life Sciences primer B and the broad range bacterial primer B357R is in italics). The PCR conditions were 5 min of initial denaturation at 95°C followed by 25 cycles of denaturation (30 s at 95°C), annealing (30 s at 52°C) and elongation (60 s at 72°C), with a final extension at 72°C for 8 min.

### PCR product purification and pyrosequencing

Each PCR product was purified by filtration and equal amounts of the four samples with different sample-specific barcode sequences were pooled. Then, the pooled DNA was isolated from a 0.8% agarose gel and purified. Purifications were carried out using the High Pure PCR Product Purification Kit (Roche). The pooled DNA was sent for pyrosequencing with primer A on an eight-lane picotiter plate on a Genome Sequencer FLX system (Roche).

### Sequence analysis

Sequences with low average quality scores (<20) and short read lengths (<200 nt) were removed. The remaining sequences were checked for potential chimeras using the chimera.slayer and the chimera.pintail tools as implemented in the mothur software package v.1.13.0 [17].

### Taxonomic affiliation

The taxonomic affiliation of partial-length sequences was determined using the Classifier tool of the Ribosomal Database Project-II (RDP) [18], [19]. This method is widely used and provides rapid taxonomic classification from domain to genus of both partial and full-length 16S rRNA gene sequences. We used a 50% bootstrap threshold, stopping the assignation at the last clear taxonomic level and leaving successive levels as unclassified (uc).

### Phylotype definition

Clustering at 98% of sequence identity was carried out using cd-hit-est [20] and the resulting phylotypes were used to study sample composition at the ‘species’ level.

### Adjustment of the number of reads in each sample to the smallest data set size

Re-sampling of the 4 samples to identical sequencing depth was done by randomly selecting reads in the fasta files using Daisy_chopper v0.6 (http://www.genomics.ceh.ac.uk/GeneSwytch/Tools.html).

### Estimation of bacterial diversity

The Shannon diversity index (H) [21], that correlates positively with taxa richness and evenness, the Chao1 richness estimator [22], and rarefaction curves, were calculated for each sample at family, genus and phylotype levels (clusters at 98% sequence identity). Diversity and richness were estimated with both the full data sets and the data sets adjusted to equal sequence number.

### Statistical comparison of sample composition

The patterns of variation in the taxonomic distributions found in our samples were explored using detrended correspondence analysis (DCA).

Diversity and richness indices and DCAs were calculated using the free-licence R package [23] and the vegan R package [24].

### Nucleotide sequence accession numbers

The non-redundant sequences from this study have been deposited in the GenBank database under accession numbers JN569361 - JN570496.

## Results

### Bacterial diversity and rarefaction analysis

**Figure 2:**
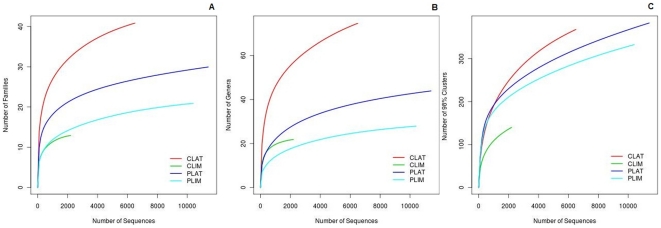
Rarefaction curves calculated at family (a), genus (b) and phylotype (clustering at 98% of identity) (c) levels. CLAT, *C. latum* larvae; CLIM, *C. limbipenne* larvae; PLAT, *C. latum* cactus medium; PLIM, *C. limbipenne* cactus medium.

We were close to completeness of the bacterial inventory at family and genus level according to the rarefaction curves (Figure 2) and the Chao1 estimator of bacterial richness (Table 1). The curves for phylotypes (at 98% identity), which do not reach the plateau, and the comparison between observed and estimated richness, indicate some of phylotypes that have been missed.

**Table 1:** Observed richness, Chao1 richness estimator (and standard error, SE), and Shannon diversity index (H) in larval guts (CLIM: *C. limbipenne*, CLAT: *C. latum*) and in decayed cactus stems (PLAT: *I. dumortieri* decayed tissues with *C. latum* larvae, PLIM: *I. dumortieri* decayed tissues with *C. imbipenne* larvae) for the full data sets and re-sampled data sets adjusted by the smallest sample size (average value and standard deviation, SD, for three replicates).

			CLAT	CLIM	PLAT	PLIM
Full	# sequences		6639	2363	11505	10572
data sets	Family	# families	41	13	30	21
		Chao1 (SE)	45 (4.84)	13 (0.73)	35 (10.17)	23 (5.29)
		Shannon H	1.87	0.91	1.48	1.20
	Genus	# genera	75	22	44	28
		Chao1 (SE)	86 (8.33)	22 (1.87)	53 (10.68)	33 (10.17)
		Shannon H	2.16	1.86	1.50	1.23
	Clusters 98%	# clusters	370	143	384	334
		Chao1 (SE)	464 (24.84)	188 (19.84)	651 (74.50)	473 (39.93)
		Shannon H	4.84	3.99	5.09	4.98
Re-sampled	# sequences		2363	2363	2363	2363
data sets	Family	# families	33 (5.51)	13	22 (1.15)	14 (1)
	(average (SD))	Chao1	65 (44.59)	13	24 (2.65)	15 (1.26)
		Shannon H	1.86 (0.01)	0.91	1.46 (0.02)	1.19 (0.02)
	Genus	# genera	59 (6.66)	22	28 (2.08)	18 (2.52)
	(average (SD))	Chao1	97 (36.84)	22	39 (15.49)	20 (3.93)
		Shannon H	2.14 (0.02)	1.86	1.48 (0.02)	1.22 (0.02)
	Clusters 98%	# clusters	263 (9.45)	143	236 (3.51)	219 (4.62)
	(average (SD))	Chao1	381 (43.01)	188	324 (21.44)	283 (33.42)
		Shannon H	4.78 (0.03)	3.99	5.02 (0.02)	4.92 (0.01)

The Shannon diversity index (Table 1), calculated at each taxonomic level (family, genus, phylotype), show the same tendency between species in larval and substrate samples: *C. latum* is more diverse than *C. limbipenne*, and PLAT is more diverse than PLIM. In a global view, *C. latum* is the most diverse sample, except at the phylotype level, where substrate samples are more diverse than *C latum*. This fact is probably due to the higher number of sequences obtained from PLAT and PLIM regarding larval samples.

As shown in Figure 2 and Table 1, the bacterial communities inhabiting both insect and cactus samples show a high level of diversity, with hundreds of different phylotypes in each sample. Among the insect samples, CLAT gut microbiota is the most complex. It displays the highest diversity indices at any of the three levels of diversity considered, whereas CLIM gut microbiota is less diverse, partly because CLIM has the lowest number of sequences. These facts could be related to the complexity of the vegetal substrates they feed on (i.e., a more diverse microbiota is expected in insects that feed on more complex substrates, composed of different polymeric substances whose degradation in anaerobic gut conditions requires a more complex microbial community).

### Bacterial distribution among samples

**Figure 3:**
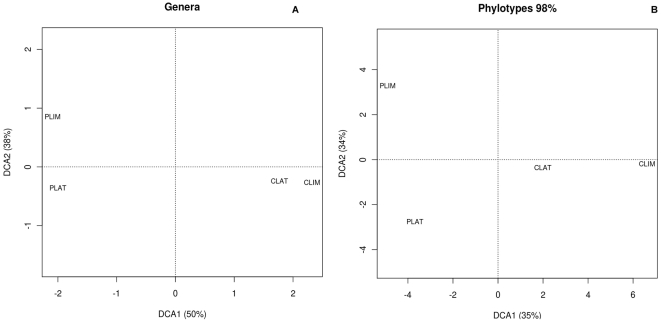
Detrended correspondence analysis based on a) genus and b) phylotype distributions. CLAT, *C. latum* larvae; CLIM, *C. limbipenne* larvae; PLAT, *C. latum* cactus medium; PLIM, *C. limbipenne* cactus medium.

DCAs indicate that the bacterial communities present in both fly species are more related to each other than to those harboured in their plant substrates (Figure 3). The first DCA axis clearly separates insects from their substrates. It explains 50% of variance at genus level, and 35% at phylotype level. The second axis separates both types of substrates, and accounts for 38% of variance at genus level, and 34% at phylotype level. However, both insects clearly harbour different communities that include species-specific sequences, as well as bacterial groups that are widespread in other Diptera analyzed so far [25] (see below). In addition, some phylotypes are shared only among larvae and the plant they feed on, whereas every sample harbours distinctive phylotypes.

An overall description of the sequences found in the analyzed samples is shown in Table 2, where a list of the taxonomic affiliation of the sequences down to genus level, together with their relative frequency in each of the 4 analyzed samples, is provided. Larva and cactus microbiota differ not only in genus composition but also in the relative frequency of shared genera. In good agreement with the diversity data discussed above, there are many genera that are only present in *C. latum,* such as the putative tethatrionate oxidyzing *Tetrathiobacter*, which accounts for almost 5% of CLAT sequences. Most of those genera belonged to the classes Actinobacteria and Alphaproteobacteria. Another distinctive feature of *C. latum* is the high prevalence of Gammaproteobacteria, most of them non-characterized below the class taxonomic level (52% of all sequences). In contrast, *C. limbipenne* is characterized by a high prevalence of Enterobacteriaceae (76% of all sequences), most of them within the *Enterobacter*, *Citrobacter* and *Pectobacterium* genera. The lactic acid bacteria *Lactobacillus* and *Leuconostoc* are the most frequently retrieved genera in the cactus tissues, although they are also found in the larval samples.

**Table 2:** Taxonomic composition of the samples (percentage of sequences belonging to each bacterial genus).

Phylum	Class	Order	Family	Genus	CLAT	CLIM	PLAT	PLIM
Acidobacteria	Acidobacteria Gp1	Acidobacteria Gp1	Acidobacteria Gp1	*Gp1*			0.0001	
Actinobacteria	Actinobacteria	Actinomycetales	Actinomycetaceae	uc Actinomycetaceae	0.0003			
			Cellulomonadaceae	*Cellulomonas*	0.0002			
			Corynebacteriaceae	*Corynebacterium*	0.0003			
			Dietziaceae	*Dietzia*	0.0020			
			Microbacteriaceae	*Agreia*	0.0002			
				*Microbacterium*	0.0002			
				uc Microbacteriaceae	0.0002			
			uc Actinomycetales	uc Actinomycetales	0.0051		0.0001	0.0001
Bacteroidetes	Bacteroidia	Bacteroidales	Bacteroidaceae	*Bacteroides*	0.0012	0.0008		
			Porphyromonadaceae	*Butyricimonas*	0.0003			
				*Dysgonomonas*	0.0434	0.0719	0.0002	0.0003
				*Parabacteroides*	0.0051		0.0067	0.0012
				*Proteiniphilum*	0.0015			
				uc Porphyromonadaceae	0.0048	0.0106		0.0001
			Prevotellaceae	*Prevotella*				0.0003
				uc Prevotellaceae				0.0017
			Rikenellaceae	*Alistipes*	0.0005	0.0013	0.0002	
			uc Bacteroidales	uc Bacteroidales				0.0023
	Flavobacteria	Flavobacteriales	Flavobacteriaceae	*Wautersiella*		0.0038	0.0002	
				uc Flavobacteriaceae	0.0002	0.0017	0.0003	
			uc Flavobacteriales	uc Flavobacteriales	0.0006			
	Sphingobacteria	Sphingobacteriales	Sphingobacteriaceae	*Parapedobacter*	0.0005		0.0003	
	uc Bacteroidetes	uc Bacteroidetes	uc Bacteroidetes	uc Bacteroidetes	0.0380	0.0013		0.0003
Cyanobacteria	Cyanobacteria	Cyanobacteria	Chloroplast	*Streptophyta*			0.0022	
Firmicutes	Bacilli	Lactobacillales	Aerococcaceae	*Facklamia*	0.0015		0.0059	
			Enterococcaceae	*Enterococcus*	0.0008	0.0030	0.0009	
				*Vagococcus*	0.0012		0.0068	0.0002
				uc Enterococcaceae	0.0003	0.0245	0.0003	
			Lactobacillaceae	*Lactobacillus*	0.0524	0.0161	0.3955	0.6222
				uc Lactobacillaceae	0.0002		0.0009	0.0031
			Leuconostocaceae	*Leuconostoc*	0.0033	0.0008	0.4075	0.0760
			Streptococcaceae	*Lactococcus*	0.0068		0.0221	
			uc Lactobacillales	uc Lactobacillales	0.0008	0.0080	0.0173	0.0501
		uc Bacilli	uc Bacilli	uc Bacilli	0.0047	0.0004	0.0694	0.1860
	Clostridia	Clostridiales	Lachnospiraceae	uc Lachnospiraceae				0.0001
			Ruminococcaceae	uc Ruminococcaceae	0.0003		0.0001	0.0003
			Veillonellaceae	*Allisonella*				0.0001
			uc Clostridiales	uc Clostridiales			0.0003	
	Erysipelotrichi	Erysipelotrichales	Erysipelotrichaceae	*Erysipelothrix*	0.0003		0.0001	
				uc Erysipelotrichaceae	0.0003		0.0010	0.0002
	uc Firmicutes	uc Firmicutes	uc Firmicutes	uc Firmicutes	0.0002		0.0142	0.0436
Proteobacteria	Alphaproteobacteria	Rhizobiales	Aurantimonadaceae	uc Aurantimonadaceae	0.0002			
			Brucellaceae	*Ochrobactrum*	0.0203			
				uc Brucellaceae	0.0003			
			Hyphomicrobiaceae	*Devosia*	0.0104			
			Xanthobacteraceae	*Xanthobacter*	0.0003			
				uc Xanthobacteraceae	0.0023			
			uc Rhizobiales	uc Rhizobiales	0.0002			
		Rhodobacterales	Rhodobacteraceae	*Haematobacter*	0.0009			
				*Ketogulonicigenium*	0.0002			
				*Paracoccus*	0.0491			
				*Rhodobacter*	0.0002			
				uc Rhodobacteraceae	0.0020			
		Rhodospirillales	Acetobacteraceae	*Acetobacter*	0.0003		0.0120	0.0076
	Betaproteobacteria	Burkholderiales	Alcaligenaceae	*Achromobacter*	0.0005			
				*Alcaligenes*	0.0054		0.0013	
				*Bordetella*	0.0008			
				*Castellaniella*	0.0015		0.0001	
				*Kerstersia*	0.0054		0.0001	
				*Pigmentiphaga*	0.0011			
				*Pusillimonas*	0.0015		0.0001	
				*Tetrathiobacter*	0.0499			
				uc Alcaligenaceae	0.0640		0.0025	0.0001
			Comamonadaceae	*Comamonas*	0.0193		0.0005	
				uc Comamonadaceae	0.0117		0.0003	
			Oxalobacteraceae	*Oxalicibacterium*	0.0039			
				uc Oxalobacteraceae	0.0002			
			uc Burkholderiales	uc Burkholderiales	0.0041		0.0006	
		uc Betaproteobacteria	uc Betaproteobacteria	uc Betaproteobacteria				0.0003
	Deltaproteobacteria	Bdellovibrionales	Bdellovibrionaceae	*Bdellovibrio*	0.0003			
	Epsilonproteobacteria	Campylobacterales	Campylobacteraceae	*Campylobacter*	0.0002			
	Gammaproteobacteria	Enterobacteriales	Enterobacteriaceae	*Citrobacter*	0.0050	0.0677		0.0007
				*Enterobacter*	0.0027	0.2603		
				*Erwinia*	0.0057	0.0025	0.0003	
				*Klebsiella*	0.0002	0.0004		
				*Morganella*	0.0002			
				*Pectobacterium*	0.0036	0.0478	0.0255	0.0019
				*Providencia*	0.0030	0.0102	0.0003	
				*Salmonella*	0.0002			
				*Serratia*				0.0001
				uc Enterobacteriaceae	0.0090	0.3737	0.0007	0.0003
		Oceanospirillales	uc Oceanospirillales	uc Oceanospirillales		0.0030		
		Pasteurellales	Pasteurellaceae	uc Pasteurellaceae	0.0170			
		Xanthomonadales	Xanthomonadaceae	*Luteimonas*	0.0009			
				*Stenotrophomonas*			0.0001	
		uc Gammaproteobacteria	uc Gammaproteobacteria	uc Gammaproteobacteria	0.5192	0.0901	0.0017	0.0007
	uc Proteobacteria	uc Proteobacteria	uc Proteobacteria	uc Proteobacteria			0.0002	
Synergistetes	Synergistia	Synergistales	Synergistaceae	*Aminiphilus*			0.0001	
Tenericutes	Mollicutes	Acholeplasmatales	Acholeplasmataceae	*Acholeplasma*			0.0010	
		uc Mollicutes	uc Mollicutes	uc Mollicutes			0.0001	
uc Bacteria	uc Bacteria	uc Bacteria	uc Bacteria	uc Bacteria	0.0002		0.0003	0.0004

CLAT: *Copestylum latum*, CLIM: *Copestylum limbipenne*, PLAT: *C. latum* cactus breeding medium, PLIM: *C. limbipenne* cactus breeding medium.

## Discussion

This is the first attempt to describe the gut bacterial communities in *Copestylum* larvae that breed in decomposed cacti. It is also the first report about bacterial species in *Isolatocereus dumortieri* (columnar cactus). Foster and Fogleman [26] reported bacteria in columnar cactus rotten tissues from *Stenocereus thurberi* (pipe cactus), *Carnegieae gigantean* (saguaro) and *Lophocereus schotii* (senita cactus). In contrast to our study, they found *Pseudomonas, Staphylococcus, Enterococcus* and *Xantomonas,* and similar to us, *Erwinia*.

*C. latum* and *C. limbipenne* samples display a high relative abundance of Gammaproteobacteria, mostly Enterobacteriaceae in *C. limbipenne.* Two enterobacterial genera (*Enterobacter* and *Klebsiella*) are found only in larval samples. Enterobacteria are heterotrophic facultative anaerobes and have frequently been found in insect microbiota using both culture and molecular techniques [27, 28 29; 30 31, 32; 33]. Some of these Enterobacteria are diazotrophs (i.e. nitrogen fixing), which would provide the insect with an obvious advantage in an environment depleted in fixed oxygen. In our case, some of the enterobacterial genera detected (*Citrobacter*, *Enterobacter*, *Erwinia*, *Klebsiella*) include diazotrophic species not present in the vegetal substrate microbiota. Given the selective advantage that the availability of a fixed nitrogen source would provide for the insect host, one could speculate that these bacteria are harboured in the larvae due to a vertical transmission, as postulated for the fruit fly [33]. In addition to these putatively nitrogen-fixing bacteria, both cacti and larvae include the pectinolytic and phytopathogenic genus *Pectobacterium*, also found in the fruit-fly.

Interestingly, other genera also involved in the nitrogen cycling have been found in association with *C. latum* larvae in a relatively high abundance. Such is the case of *Paracoccus* and *Comamonas*, which include some denitrifying species [25].

Compared to previously published studies on insect microbial diversity, there are some remarkable differences. An example is the absence of bacteria from the genera *Spiroplasma*, *Wolbachia* and *Bacillus*, frequently found in association with other insects [27]; [34]; [35]; [36]; [30]; [29], but absent from both *C. latum* and *C. limbipenne* larva. The acetic acid bacteria *Acetobacter*, which has recently been described as a newly emerging symbiont of insects, is mostly absent from the CLAT and CLIM larvae, although it is relatively abundant in the cactus tissue colonized by CLAT. Conversely, these species harbour bacteria that have not previously been found associated with insects, such as *Dysgonomonas*, *Ochrobactrum* and *Devosia*, for example [25]. Lactic acid bacteria that ferment sugars are found frequently as plant-commensal microbiota and also as part of insect-associated bacteria, where it has been speculated that they play a role in the larval digestive tract [27].

The results obtained here indicate that the insect microbiota is not the same as that found in its corresponding vegetable substrate, since there are many bacterial groups in the insects that have not been found in their substrates. However, substrates could act as a reservoir for newly acquired species, which can eventually become part of the commensal community. Furthermore, the gut bacterial community in these insects could be partially inherited by vertical transmission from mother to offspring. Thus, in the fruit fly-bacteria association, Ben-Yosef *et al*. [33] found that the microbiota is vertically transmitted and colonizes the plant surface after hatching. According to these authors, the larva would carry a “survival pack” of bacteria, including nitrogen fixing and pectinolytic genera, which would help in the first stages of plant colonization. In fact, as discussed above, CLAT and CLIM also harbour putatively pectinolytic and diazotrophic Enterobacteriaceae. Other factors shaping the specific commensal/mutualistic bacteria, such as the host innate immunity and evolutionary history-events (constrains, isolation, horizontal transmission, etc.), cannot be ruled out. The gut microbiota of the two Copestylum species is relatively similar, as one could expect in two phylogenetically related insect species living in similar ecological niches. On the other hand, the differences in the microbiota between the two substrates should correspond to the bacterial succession that is taking place during the decomposition process of the cactus.

Another factor that could affect the studied communities is the presence of plant allelochemicals that could restrict the growth of some cactophilic yeast and bacterial species [37]. For instance, Starmer *et al.* [38] recognized that some species of yeast were inhibited by some triterpene glycosides found in some columnar cacti. One possibility is that some bacteria are better adapted to the cactus necrotic niche and are more tolerant to potential toxic secondary plant compounds, because many columnar cacti have triterpene glycosides and isoquinone alkaloids [39]. Kinoshita [40] found one triterpenoid saponin called dumortierninoside A, but the role of this triterpene in dipteran-cactus relationships is unknown.

Finally, the availability of specific nutrients and dipteran adaptations for cactus species can be related to the specificity in the microbiota. It has been shown that some Drosophilids have the ability to metabolize volatiles, such as ethanol vapor, as an adaptation for survival in volatile-rich columnar cactus rots [41]. We can speculate that this ability is provided by the microbiota. These evolutionary trends have not yet been proved in *Copestylum* species. Moreover, this information is necessary to understand the evolution of Dipteran species in cactus necrosis. In this research, we only analyze decomposed stems of *I. dumortieri* cactus, but central Mexican scrublands have other cactus species used as breeding places for *Copestylum* larvae [4], [5], [8], [3], [6], [7]. Therefore, the complexity of this system needs to be investigated as in the case of the cactus-Drosophila-microorganism system. More details about the differences in the bacterial communities from the first decayed cactus stems to rotten tissues and the differences with other feeding strategies (e.g. straining larvae) will be of interest to understand the role of bacteria in the decomposition process and in the colonization of syrphid species.
